# Anti-Tubercular Properties of 4-Amino-5-(4-Fluoro-3- Phenoxyphenyl)-4*H*-1,2,4-Triazole-3-Thiol and Its Schiff Bases: Computational Input and Molecular Dynamics

**DOI:** 10.3390/antibiotics9090559

**Published:** 2020-08-31

**Authors:** Katharigatta N. Venugopala, Mahmoud Kandeel, Melendhran Pillay, Pran Kishore Deb, Hassan H. Abdallah, Mohamad Fawzi Mahomoodally, Deepak Chopra

**Affiliations:** 1 Department of Pharmaceutical Sciences, College of Clinical Pharmacy, King Faisal University, Al-Ahsa 31982, Saudi Arabia; 2 Department of Biotechnology and Food Technology, Durban University of Technology, Durban 4001, South Africa; 3 Department of Biomedical Sciences, College of Veterinary Medicine, King Faisal University, Al-Ahsa 31982, Saudi Arabia; mkandeel@kfu.edu.sa; 4 Department of Pharmacology, Faculty of Veterinary Medicine, Kafrelsheikh University, Kafrelsheikh 33516, Egypt; 5 Department of Microbiology, National Health Laboratory Services, KZN Academic Complex, Inkosi Albert Luthuli Central Hospital, Durban 4001, South Africa; melendhra.pillay@nhls.ac.za; 6 Department of Pharmaceutical Sciences, Faculty of Pharmacy, Philadelphia University, P.O. Box 1, Amman 19392, Jordan; prankishore1@gmail.com; 7 Chemistry Department, College of Education, Salahaddin University, Erbil 44001, Iraq; hassan.abdullah@su.edu.krd; 8 Institute of Research and Development, Duy Tan University, Da Nang 550000, Vietnam; f.mahomoodally@uom.ac.mu; 9 Department of Health Sciences, Faculty of Science, University of Mauritius, Réduit 80835, Mauritius; 10 Department of Chemistry, Indian Institute of Science Education and Research Bhopal, Bhopal By-Pass Road, Bhauri, Bhopal 462 066, Madhya Pradesh, India; dchopra@iiserb.ac.in

**Keywords:** *Mycobacterium tuberculosis*, triazole analogues, molecular dynamics studies, β-ketoacyl carrier protein synthase III (FABH), β-ketoacyl ACP synthase I (KasA), CYP121, dihydrofolate reductase, enoyl-acyl carrier protein reductase, *N*-acetylglucosamine-1-phosphate uridyltransferase, multi-drug-resistant tuberculosis

## Abstract

In the present investigation, the parent compound 4-amino-5-(4-fluoro-3-phenoxyphenyl)-4*H*-1,2,4-triazole-3-thiol (**1**) and its Schiff bases **2**, **3**, and **4** were subjected to whole-cell anti-TB against H37Rv and multi-drug-resistant (MDR) strains of *Mycobacterium tuberculosis* (MTB) by resazurin microtiter assay (REMA) plate method. Test compound **1** exhibited promising anti-TB activity against H37Rv and MDR strains of MTB at 5.5 µg/mL and 11 µg/mL, respectively. An attempt to identify the suitable molecular target for compound **1** was performed using a set of triazole thiol cellular targets, including β-ketoacyl carrier protein synthase III (FABH), β-ketoacyl ACP synthase I (KasA), CYP121, dihydrofolate reductase, enoyl-acyl carrier protein reductase, and *N*-acetylglucosamine-1-phosphate uridyltransferase. MTB β-ketoacyl ACP synthase I (KasA) was identified as the cellular target for the promising anti-TB parent compound **1** via docking and molecular dynamics simulation. MM(GB/PB)SA binding free energy calculation revealed stronger binding of compound **1** compared with KasA standard inhibitor thiolactomycin (TLM). The inhibitory mechanism of test compound **1** involves the formation of hydrogen bonding with the catalytic histidine residues, and it also impedes access of fatty-acid substrates to the active site through interference with α5–α6 helix movement. Test compound **1**-specific structural changes at the ALA274–ALA281 loop might be the contributing factor underlying the stronger anti-TB effect of compound **1** when compared with TLM, as it tends to adopt a closed conformation for the access of malonyl substrate to its binding site.

## 1. Introduction

**Figure 1:**
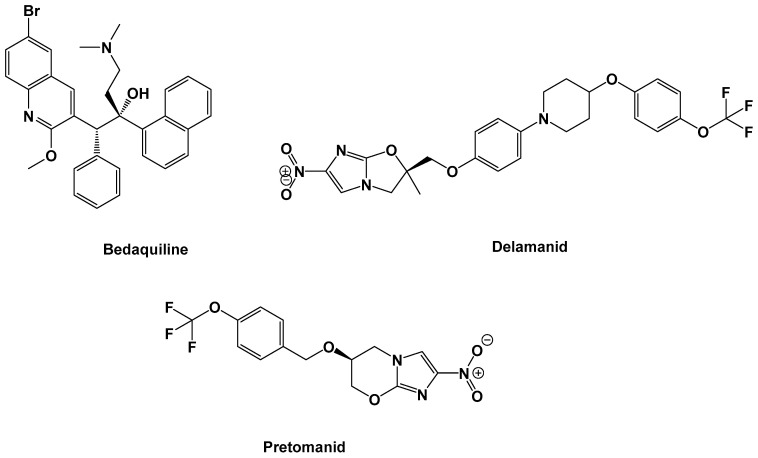
Chemical structure of recently clinically approved anti-tuberculosis (TB) drugs.

*Mycobacterium tuberculosis* (MTB) is the bacterial species underlying tuberculosis (TB) infection. TB is one of the top10 causes of death globally. In 2018, TB was implicated in ~1.5 million and 251,000 deaths among HIV-negative and HIV-positive people, respectively [1]. The increasing prevalence of TB in the form of multi-drug-resistant (MDR)-TB [2] and extensively drug-resistant (XDR)-TB [3] has triggered the necessity for more effective therapeutic regimens with fewer side effects. Treating and managing MDR-TB and XDR-TB has proven to be more challenging, as second-line drugs have largely become less effective [4]. This problem has been made worse by the emergence of totally drug-resistant (TDR) strains of MTB [5], which do not respond to anti-TB drug treatments. Based on the last 40 years of academic and pharmaceutical industry inventions, only bedaquiline was the first novel anti-TB drug permitted by the United States Food and Drug Administration (US FDA) authority in December 2012, for the treatment of MDR-TB, [6] while delamanid was the second anti-TB agent to be approved by the European Medicines Agency in late 2013 [7] and pretomanid was the third drug to be approved by the US FDA in August 2019 [8,9] (Figure 1).

Triazole pharmacophore with various functional groups/substitutions has been reported for its promising anti-TB [10,11,12,13,14,15,16,17,18,19,20,21], antiviral [22], antibacterial [23,24], antifungal [25,26], antioxidant [27,28,29,30], and antiglycation properties [31]. In addition, it also serves as an opener of Ca(^2+^)-activated potassium (maxi-K) channels [32] and demonstrates molluscicidal [33], hypoglycemic [34], antihypertensive and blood platelet aggregation inhibition [35] activities. Schiff bases of triazole compounds are also reported for their potential anti-TB agents [36]. Based on the above observations, and in continuation to our anti-TB drug discovery program, it was envisaged that the triazole parent compound 4-amino-5-(4-fluoro-3-phenoxyphenyl)-4*H*-1,2,4-triazole-3-thiol (**1**) and its Schiff bases **2**, **3** and **4** be tested for their whole-cell anti-TB activity against H37Rv and MDR strains of *M. tuberculosis*, which is resistant to treatment with rifampicin and isoniazid (1 and 0.2 µg/mL, respectively), as determined by resazurin microtiter assay (REMA) plate method.

**Figure 2:**
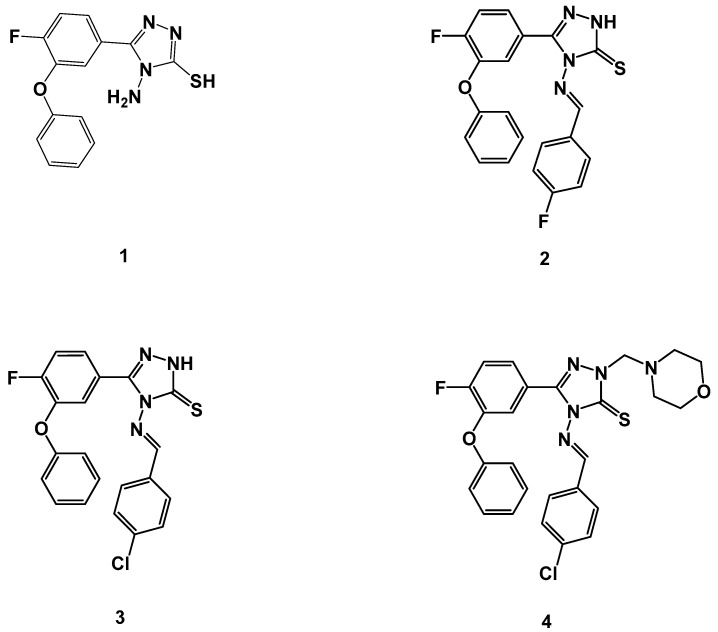
Molecular structures of triazole parent compound **1**, and its Schiff bases **2**, **3**, and **4** were tested for their in vitro anti-TB activity against H37Rv and multi-drug-resistant (MDR) strains of *Mycobacterium tuberculosis* (MTB).

Due to the urgent call for novel scaffolds as anti-TB agents, we have recently launched a medicinal chemistry program aimed at developing novel, natural [37], cyclic depsipeptides [38] and heterocyclic scaffolds as potential anti-TB agents [39,40,41,42,43,44,45,46,47]. A new scaffold of triazole thiols produced antibacterial effects by targeting folate synthesis via bacterial dihydrofolate reductase [48]. Furthermore, 5-(pyrazin-2-yl)-4*H*-1,2,4-triazole-3-thiol derivatives showed considerable antibacterial activity by targeting bacterial β-ketoacyl-acyl carrier protein synthase III (FabH) [49]. Additionally, β-ketoacyl ACP synthase I (KasA) constitutes an important drug target against MTB. Several triazole derivatives showed a significant correlation between anti-TB activity and the binding potency with KasA [36,50] Triazole derivatives have been associated with strong anti-TB activity [10,11,12,13,14,15,16,17,18,19,20,21]. Previous reports indicated that several molecular targets could be modulated by triazole-based compounds [51]; the 3-aryl-5-(alkyl-thio)-1*H*-1,2,4-triazole derivative produced anti-TB effects at a concentration of 0.03 µg/mL [16]. A significant correlation was estimated between anti-TB efficacy and binding score with MTB CYP121 [16,52]. A new triazole thiol derivative was able to inhibit the growth of MTB in the low micromolar range. Computational studies revealed strong binding with enoyl-acyl carrier protein reductase [53]. Heterocyclic hybrids of triazole inhibited MTB after binding with enoyl reductase (inhA) [54]. A virtual screening study against *Mycobacterium N*-acetylglucosamine -1-phosphate uridyltransferase discovered several compounds with a triazole nucleus and produced 20–60% inhibition of the enzymatic activity [55]. The previously mentioned targets were used in computational studies to discover the potential target of our synthetic test compounds. In the present investigation, we intended to explore the impact of the functionalization on the triazole nucleus, particularly as related to anti-TB activity against H37Rv and MDR strains of MTB (Figure 2). A whole-cell anti-TB screening process will help to identify the key substituent responsible for the activity, thereby uncovering potential molecular target(s) through a computational docking study.

## 2. Results and Discussion

### 2.1. Anti-Tubercular Activity

The concentrations of the test compounds being considered for anti-TB screening were 0.2–32 μg/mL against H37Rv and MDR strains of MTB. The MDR isolates were resistant to first-line treatments, including rifampicin at 1 µg/mL and isoniazid at 0.2 µg/mL. Of the four compounds examined for their anti-TB activity, the most promising was parent compound **1** at 5.5 and 11 µg/mL, respectively, against H37Rv and MDR strains of MTB. Test compounds **2**, **3**, and **4** were active only against H37Rv; however, they failed to show anti-TB activity against MDR strains up to concentrations of 32 µg/mL. Compounds **2** and **4** revealed similar anti-TB activities at concentrations of 2 µg/mL against H37Rv strains of MTB. In vitro whole-cell anti-TB results of title compound **1** and its Schiff bases **2**, **3**, and **4** against H37Rv and MDR strains of MTB are tabulated in Table 1.

### 2.2. Toxicity Studies

The anti-TB compounds from Table 2 were evaluated for safety studies via MTT assay. Overall, compound **1** exhibited no toxicity up to 450 µg/mL across peripheral blood mononuclear (PBM) cell lines.

### 2.3. Computational Studies

Molecular docking followed by MD simulation was used to identify the potential target of the detected anti-TB activity of test compound **1**. For this purpose, compound **1** was docked into several structures with confirmed interactions with triazole derivatives. These targets included various metabolic pathways and carrier proteins, including fatty-acid metabolism, cytochrome enzyme, and cell-wall biosynthesis. By using template construction followed by docking, 10 poses were generated for compound **1** with the protein structures shown in Table 3. The poses were ranked by their lowest docking score and root mean square deviation (RMSD). The best pose was selected based on its lowest docking score and RMSD as compared with the bound substrate. Then, the relative docking score was calculated according to Equation (1). Based on the relative docking score, KasA was predicted as the most important target for compound **1** due to the highest relative docking score. For the MD simulation experiment, the MTB KasA structure bound with test compound **1** was generated from the docking study. During the MD experiment, the changes in MTB KasA-compound **1** were compared with ApoKasA and KasA bound with its co-crystal inhibitor ligand TLM.

### 2.4. The Binding Interactions of TLM and the Most Promising Test Compound **1** (4-Amino-5-(4-Fluoro-3-Phenoxyphenyl)-4H-1,2,4-Triazole-3-Thiol)

**Figure 3:**
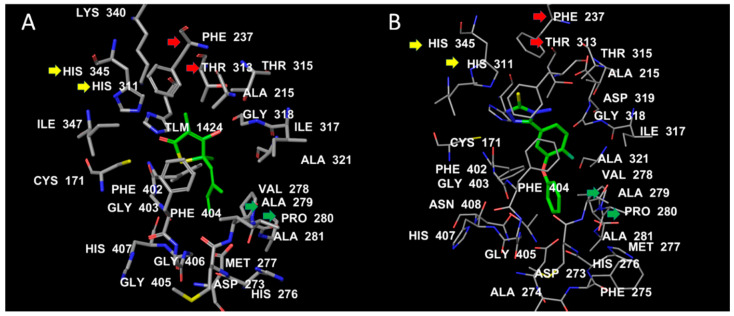
The binding interactions of TLM (**A**) and test compound **1** (**B**) with MTB KasA. The ligand hydrocarbons are displayed in green, while the protein residues are colored by atom type, where carbons are white, nitrogen is blue, and oxygen is red. The labels of the active-site residues are displayed by residue number.

**Figure 4:**
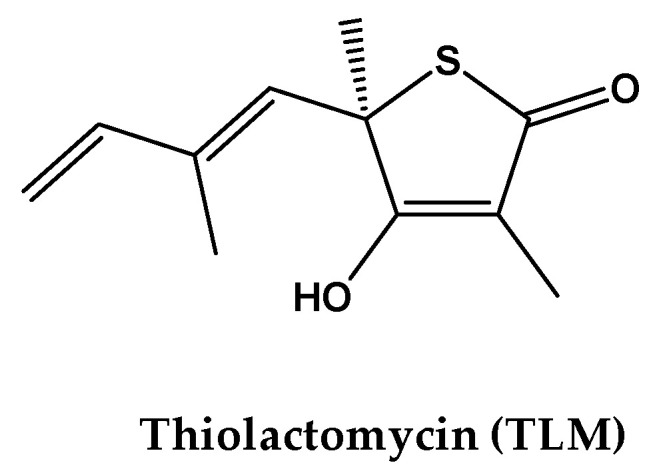
Chemical structure of the co-crystal ligand thiolactomycin (TLM) of β-ketoacyl ACP synthase I (KasA).

Both TLM and test compound **1** showed conserved interactions with the two catalytic histidine residues HIS311 and HIS345 (yellow arrows). The C10 of TLM corresponds to SH in compound **1**, which fills a hydrophobic cavity formed by PHE237 and THR313 (red arrows; Figure 3). The isoprene chain of TLM engages in hydrophobic interactions with ALA279 and PRO280 (green arrows). At a similar position, the benzene ring of compound **1** is bulkier than the isoprene chain and makes further hydrophobic contact with the flexible loop ALA274–ALA281. This might be an additional stabilizing factor for compound **1** when compared with TLM (Figure 4).

To elaborate the conformational stability, the deviations and fluctuations of KasA structure either alone (blue) or in complex with TLM (orange) or compound **1** (grey) were traced by recording all atoms Cα-RMSD. At equilibrium, the observed root mean square deviation (RMSD) for ApoKasA was at 3.4 Å, while it was 2.2 Å for both TLM and compound **1** complexes. However, the compound **1** complex showed lower divergence. In addition, the KasA-compound **1** complex was almost stable and sowing gradual increase in RMSD during the first 80 ns, while KasA-TLM complex showed an increase in RMSD in the first 100 ns. The TLM complex showed fluctuations and variations of RMSD values that are higher than the compound **1** complex. This highlights the improved stability of the KasA complex with compound **1** over TLM. The RMSD of the α-carbon atom of both KasA-TLM and KasA-compound **1** showed lower values when compared with ApoKasA. This indicates that both TLM and compound **1** complexes produced low conformational changes in the protein backbone as compared with the ApoKasA. Binding of both TLM and parent compound **1** significantly stabilized MTB KasA by showing lower RMSD values throughout the simulation time, as compared with ApoKasA. TLM and compound **1** showed almost similar profiles, although KasA-compound **1** showed rapid stability and lower RMSD at the start of the simulation. The generalized lower average of RMSD for KasA-compound **1** indicates greater stability of KasA when bound with compound **1**, as compared with TLM. The average simulation RMSD was 2.97 ± 0.39, 2.41 ± 0.27, and 2.11 + 0.26 for ApoKasA, KasATLM, and KasA-compound **1**, respectively. This indicates the improved complex stability of compound **1** over TLM.

**Figure 5:**
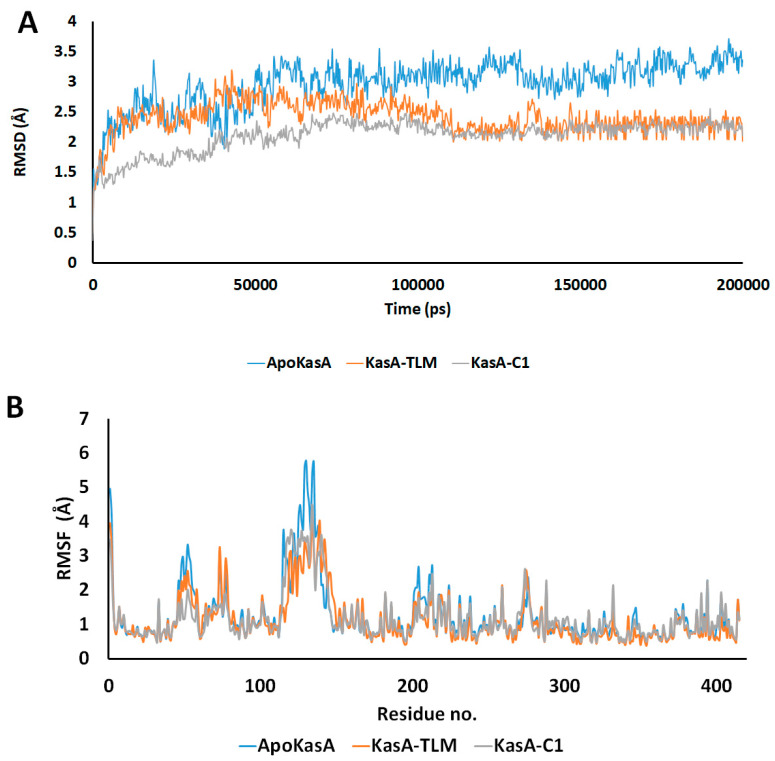
(**A**) Time dependent RMSD of the α-carbon atom for ApoKasA, KasA bound with TLM, and KasA bound with the compound **1**. The simulation was run for 200 ns. (**B**) Per-residue changes in root mean square deviation (RMSD) during 200 ns of simulation. The *X*-axis represents the residue number and the *Y*-axis is the RMSD of the α-carbon atom. The color blue, orange and grey represents the structures of ApoKasA, KasA bound with TLM, and KasA bound with the compound **1**, respectively.

**Figure 6:**
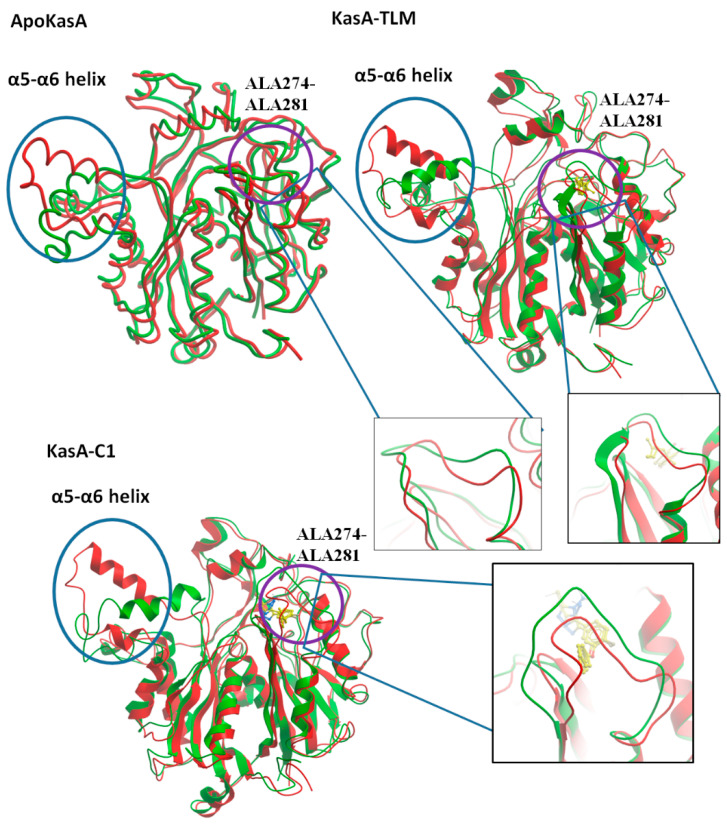
Structure alignment of MTB ApoKasA, KasA-TLM, and KasA-compound **1** (KasA-C1) before (red) and after (green) MD simulation. Two major areas of noticeable RMSD changes are the α5–α6 helix (encircled by blue circles) and ALA274–ALA281 (encircled by violet circles) and are further represented by additional insets.

Per-residue root mean square fluctuation (RMSF) was recorded to assess the fluctuations of the residues during KasA free form or in complex with TLM or compound **1**. RMSF values revealed two major sites of differences among the checked structures. The first is the α5–α6 helix (residues 115–147), which showed the highest RMSF value in ApoKasA (Figure 5B and Figure 6). The changes in the RMSF of the α5–α6 helix did not show significant differences between KasA-TLM and KasA-compound **1**. However, it was at least 2 Å lower than ApoKasA. It was found that α5–α6 helix flexibility is important for KasA activity and controls the access of the growing lipid substrates to the active site [59]. The binding of either TLM or compound **1** resulted in a lower RMSD of the α5–α6 helix. This might be the major contributing factor to their inhibitory properties against KasA. The lower flexibility of α5–α6 will provide steric hindrance for large-sized fatty-acid substrates and will inhibit enzyme activity.

The second site that exhibited important changes in RMSD was the flexible loop ALA274–ALA281 (Figure 6), which bore specific changes with the binding of compound **1** only. There were little differences between ApoKasA and KasA-TLM at this position (Figure 5B and Figure 6). In contrast, KasA-compound **1** showed a high RMSD and marked the reorientation of this loop toward the active site. A 15 ns MD simulation of MTB KasA showed marked flexibility of the ALA274–ALA281 loop, which constituted part of the entrance to the malonyl binding site [64]. The initially resolved conformation of this loop in the crystal structure adopted closed conformation, and after MD simulation, it adopted open conformation by moving away from the binding site to permit the entrance of the malonyl substrate [64]. In this study, compound **1** induced significant changes in the ALA274–ALA281 loop by adopting a closer position to the malonyl-binding site. Thus, compound **1** may interfere with malonyl binding by closing its site via inward movement of the ALA274–ALA281 loop.

MM(GB/PB)SA was used to calculate the binding free energy (Table 4). The results suggest that compound **1** has lower free energy as compared with TLM. The calculated free energy and the observed molecular dynamic changes suggest strong binding of compound **1** with mycobacterial CasA.

The cell wall of MTB is rich in long-chain lipids, which render it resistant to treatment. KasA is an important enzyme in bacterial type II fatty-acid synthesis pathways (FASII), which are required for fatty-acid elongation of up to 56 carbons and can aid in the formation of mycolic acids [59]. TLM is a natural product from *Nocardia* spp. Moreover, it showed strong anti-TB activity against saprophytic and virulent strains of MTB [65,66]. Complete inhibitory action against the growth of *M. smegmatis* mc2155 and MTB Erdman was detected at 75 µg/mL and 25 µg/mL, respectively [65]. In our study, compound **1** showed a stronger anti-TB effect that was 2–7-fold higher than TLM. Test compound **1** inhibited the growth of MTB H37Rv and MDR MTB at 5.5 µg/mL and 11 µg/mL, respectively. The improved anti-TB activity of test compound **1** over TLM was expected to be implemented by its associated MD changes in KasA structure, where the conserved interaction with the catalytic histidines, and the lowered flexibility of α5–α6 helix suggests the anti-TB effects of both of TLM and compound **1**. Additional changes in the ALA274–ALA281 loop might contribute to the superiority of compound **1** in terms of its anti-TB properties.

In the efforts of finding new inhibitors against MDR-MTB, several studies were implemented worldwide. A high throughput screening study comprising 45,000 compounds discovered a set of disubstituted oxazole with potent activity against MDR-MTB with potencies of 4–64 µg/mL [67]. Two rifabutin analogs were recently introduced as potential hopeful anti-MDR-MTB agents owing to their inhibitory effect on MDR-MTB in the range of 6–8 µg/mL [68]. The potent fluoroquinolone anti-TB ciprofloxacin showed MIC value >64 µg/mL in MDR-MTB strains. Further modifications of ciprofloxacin by several 1,2,3-triazole-isatin hybrids improved the parent compound efficiency to be 16 µg/mL [69]. An interesting feature of compound **1** in this study is its effect on MDR-MTB with high potency against rifampin and isoniazid multidrug-resistant MTB strain. The experimental and computational competency of this compound renders it as a good lead for future drug discovery studies.

**Figure 7:**
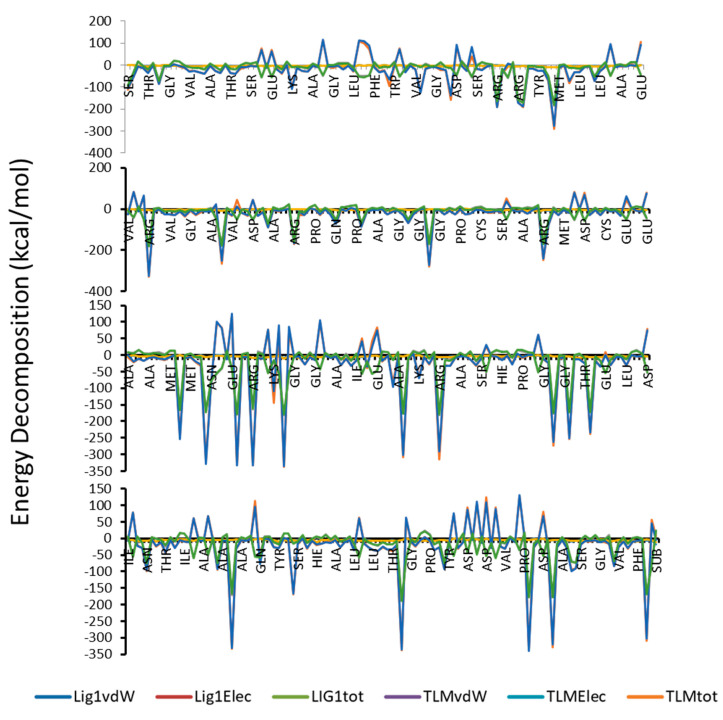
Total Energy decomposition, van der Waals and electrostatic energy (kcal/mol) for the complexes of compound **1** and TLM with protein.

It is well known that, MM(GB/PB)SA program is producing more accurate binding energy in comparison with other scoring programs like docking. As shown in Table 4 and Figure 7, compound **1** has higher binding free energy as compared to the TLM, and that was supported by the calculated values of entropy. In addition, van der Waals forces represent the favorable component and the dominant forces amongst the other forces.

## 3. Materials and Methods

### 3.1. Chemistry

The synthesis and structural elucidation of the title compounds **1**, **2**, **3**, and **4** were previously reported by our group, following a synthetic protocol [56,57]. The chemical structure of the parent compound 4-amino-5-(4-fluoro-3-phenoxyphenyl)-4*H*-1,2,4-triazole-3-thiol (**1**) and its Schiff bases **2**, **3**, and **4** are listed in Figure 2. The partition coefficient and IUPAC nomenclature of test compounds **1**–**4** are tabulated in Table 2.

### 3.2. Anti-Tubercular Activity

#### 3.2.1. Resazurin Microplate Assay (REMA)

Anti-TB screening of test compounds **1**–**3** and **4** (Figure 2) was performed using the colorimetric REMA plate approach [41,70].

#### 3.2.2. Determination of the Minimum Inhibitory Concentration (MIC)

All test compounds (**1**–**3** and **4**) were assessed using the agar incorporation approach, which was performed three times, and targeted in H37Rv and MDR-TB strains (isoniazid, 0.2 µg/mL; rifampicin, >1.0 µg/mL). MIC determination was then carried out with some modifications [71]. A Level II Biosafety Laboratory was used to carry out this experiment. MTB reference strain H37Rv (American Type Culture Collection [ATCC], Manassas, VA, USA: 25177) and MDR-TB were cultured in Middlebrook 7H11 medium for a total of 3 weeks [72]. The strain was supplemented with OADC (0.005%, *v/v*, oleic acid; 0.2%, *w/v*, glucose; 0.085%, *w/v*, NaCl; 0.02%, *v/v*, catalase; and 0.5%, 171 *w/v*, bovine serum albumin [BSA]), and incubated at a temperature of 37 °C. Fresh cultures were used to prepare a standardized inoculum in a sterile tube (5 mm in diameter) containing 0.05% Tween 80 and 4.5 mL of phosphate buffer for vortexing. The bacterial supernatant was then standardized to McFarland Number 1 with water, resulting in a bacterial concentration ~1 × 10^7^ cfu/mL. The bacterial suspension was then diluted with water, after which a total of 100 µL of the dilution was placed onto Middlebrook 7H10 agar plates with drug doses ranging from 8–0.125 µg/mL (to begin, 8 µg/mL of the drug was dissolved in distilled water and diluted twofold to achieve the desired concentration prior to being added to the agar medium). The MICs of the drugs (i.e., the concentration that inhibited >1% of the organism’s growth when compared with controls) were obtained 3 weeks following incubation. Table 2 presents the anti-TB results when compared with H37Rv (ATCC: 25177), MDR-MTB, and XDR-MTB.

#### 3.2.3. Safety Studies

Title compound **1**, which exhibited anti-TB activity against H37Rv and MDR strains of MTB, underwent safety studies by 3-(4,5-dimethylthiazol-2-yl)-2,5-diphenyltetrazolium bromide (MTT) assay. The MTT cytotoxicity assay evaluated the cytotoxic effects of compound **1,** according to the described protocol [73].

### 3.3. Computational Studies

#### 3.3.1. Dataset Construction

Triazole thiol derivatives are associated with strong antibacterial and anti-TB actions [41,49,65,74]. In order to identify the potential target of our newly synthesized parent compound **1** and its Schiff bases **2**, **3**, and **4**, a dataset was constructed comprising the most common molecular targets for triazole derivatives from the previously published literature [58,59,60,61,62,63]. The 3D-Crystal structures of target proteins and their co-crystal structures (Figure 3) were procured from the Protein Data Bank (PDB) as shown in Table 3.

#### 3.3.2. Preparation of Structures, Compound Files, and Molecular Docking

As described in Table 3, the 3D crystal structures for each molecular target were retrieved from PDB (www.rcsb.org). Each structure was checked for errors and optimized, and any errors were corrected. Side-chain errors and missing atoms were corrected. Water and all other non-relevant co-factors were removed from the binding space; then, charges and non-polar hydrogens were added. The substrates of each target were kept in the structure, followed by energy minimization. Docking was carried out by Molegro Virtual Docker 5.5 software using the MolDock scoring system. At first, the docking template was constructed using the template docking option in the Molegro software. The substrate for each molecular target was used to set up and conclude the molecular template interactions. Template docking was followed by a docking run to generate 10 poses showing the highest binding scores. Validity of the docking protocol was confirmed by redocking the co-crystalized ligands. The results showed a low root-mean-square deviation (RMSD) and accurate docking conformations. Compounds were ranked according to their docking score, and the relative docking score was calculated according to the following equation:

(1) $$\mathrm{Relative}\;\mathrm{docking}\;\mathrm{score}=\frac{\mathrm{Docking}\;\mathrm{score}\;\mathrm{of}\;\mathrm{compound}}{\mathrm{Docking}\;\mathrm{score}\;\mathrm{of}\;\mathrm{the}\;\mathrm{co}-\mathrm{crystalized}\;\mathrm{compound}}$$

#### 3.3.3. Molecular Dynamic (MD) Simulation

Although docking is a gold standard in understanding the drug–receptor binding interactions, there could be some limitations depending on docking alone to conclude the molecular forces associated with ligands binding. MD simulation is a powerful technique to analyze the receptor binding patterns, the contribution of solvent, and the dynamical aspects associated with ligands recognition. A combination of both docking and MD simulation can give a full molecular analysis in the drug discovery and optimization process. For this purpose, after molecular docking, we ran an expensive MD simulation of 200 ns to obtain a comprehensive insight into compounds-receptor complexes stability, the substructural changes in the mycobacterial protein in response to ligands binding as well as the predicted binding energy and its contributing forces.

MD simulation was used to elucidate the stability and sub-structural changes associated with the binding of test compound **1** with MTB KasA and to compare these properties with ApoKasA (PDB ID 2WGD) and KasA bound with a standard inhibitor (TLM, PDB ID 2WGE; Figure 3). The structure of KasA with the lowest energy pose of compound **1** was used in the MD studies [75]. An AMBER14 force field was implemented in YASARA (version 14.12.2). A particle-mesh Ewald algorithm was set for periodic boundary conditions and to calculate the long-range electrostatic force [76]. The simulation was run under isothermal and isobaric conditions (NPT ensemble). The protein was immersed in a simulation box filled with water at a density of 0.997 g/mL with a size of 10Å around the protein. The temperature was set to 298 K. Then, sodium chloride was added to replace the water to yield a total sodium chloride concentration of 0.9%. Initial energy minimization was carried out using the steepest descent method. The distance between the protein and the cell wall was set to 10Å. The time step was set to 2 fs; following this, simulation snapshots were collected every 100 ps. The average energy-minimized structure was used to compare the interactions of TLM (Figure 3) and test compound **1** (Figure 2) with *M. tuberculosis* KasA. Molecular dynamics was run by a predefined macro implemented in YASARA software (MD_runfast.mcr). Trajectories were recorded and analyzed by MD_analyse.mcr.

#### 3.3.4. MM(GB/PB)SA Calculation

MM(GB/PB)SA [77] was used to calculate the binding free energy, entropy, and full energy decomposition for the complexes of compound **1** and the control ligand TLM. Two hundred snapshots from the last 2 ns of the MD simulation were used to calculate the binding free energy and entropy.

## 4. Conclusions

The title compound 4-amino-5-(4-fluoro-3-phenoxyphenyl)-4*H*-1,2,4-triazole-3-thiol (**1**) has emerged as a promising anti-TB agent against H37Rv and MDR strains of MTB at concentrations of 5.5 and 11 µg/mL, respectively. This study has established preliminary data that could be considered when preparing various derivatives to achieve better triazolyl anti-TB agents, as this compound exhibited no toxicity up to 450 µg/mL. By performing computational studies, it was confirmed that compound **1** is expected to inhibit KasA and interfere with the synthesis of long-chain fatty acids, which comprise the cell wall in *Mycobacterium tuberculosis*. A comparison of compound **1** with TLM, a strong KasA inhibitor, revealed conserved interactions at the binding site and structural rearrangements during MD simulation that favored the stronger antimycobacterial actions of compound **1**.

## Figures and Tables

**Table 1:** In vitro anti-TB activity of compound **1** and its Schiff bases **2**, **3**, and **4** against H37Rv and MDR strains of MTB.

Compound Code ^a^	MIC (µg/mL)	
	H37Rv	MDR-MTB ^b^
1	5.5	11
2	20	NA
3	11	NA
4	20	NA

Note: MIC, minimum inhibitory concentration. ^a^ The synthesis and characterization of test compounds **1**–**4** are detailed in our previous reports [56,57]. ^b^ These isolates were resistant to rifampicin (1 µg/mL) and isoniazid (0.2 µg/mL). NA, not active (the concentration considered for screening was 0.2–32 µg/mL).

**Table 2:** IUPAC and cLog*P* of the title compounds used for whole-cell anti-TB screening against H37Rv and MDR strains of MTB.

Compound Code	Mol Formula (Mol Weight)	IUPAC	cLog*P*
1	C_14_H_11_FN_4_OS (302)	4-Amino-5-(4-fluoro-3-phenoxyphenyl)-4*H*-1,2,4-triazole-3-thiol	3.9987
2	C_21_H_14_F_2_N_4_OS (408)	(*E*)-5-(4-Fluoro-3-phenoxyphenyl)-4-((4-fluorobenzylidene)amino)-2,4-dihydro-3*H*-1,2,4-triazole-3-thione	5.2660
3	C_21_H_14_ClFN_4_OS (425)	(*E*)-4-((4-Chlorobenzylidene)amino)-5-(4-fluoro-3-phenoxy phenyl)-2,4-dihydro-3*H*-1,2,4-triazole-3-thione	5.8360
4	C_26_H_23_ClFN_5_O_2_S (524)	(*E*)-4-((4-Chlorobenzylidene)amino)-5-(4-fluoro-3-phenoxyphenyl)-2-(morpholinomethyl)-2,4-dihydro-3*H*-1,2,4-triazole-3-thione	6.7721

**Table 3:** The molecular targets used in the computational study to identify the potential target for test the compound **1**.

Molecular Target Name	PDB ID	Test Compound 1 Docking Score	Co-Crystalized Substrate Docking Score	Relative Docking Score	Reference
β-ketoacyl carrier protein synthase III (FABH)	2QX1	−227	−338	0.67	[58]
β-ketoacyl ACP synthase I (KasA)	2WGE	−644	−508	1.27	[59]
CYP121	5WP2	−413	−455	0.9	[60]
Dihydrofolate reductase	1DF7	−411	−609	0.68	[61]
Enoyl-acyl carrier protein reductase	4COD	−434	−600	0.72	[62]
*N*-acetylglucosamine-1-phosphate uridyltransferase	2QKX	−470	−478	0.99	[63]

**Table 4:** MM(GB/PB)SA binding free energy and entropy (kcal/mol) for compound **1** and TLM.

Compound Codes	Binding Free Energy		Entropy
	MMGBSA	MMPBSA	
Compound **1**	−45.553 (2.15) *	−29.096 (2.95)	−33.613
TLM	−28.042 (1.17)	−21.0636 (1.98)	−31.732

* standard deviation.
